# From Low-Cost *Miscanthus × giganteus* to Valuable Bacterial Nanocellulose: A Complete Technological Cycle

**DOI:** 10.3390/polym17212890

**Published:** 2025-10-29

**Authors:** Nadezhda A. Shavyrkina, Evgenia K. Gladysheva, Anastasia A. Zenkova, Ekaterina A. Skiba

**Affiliations:** 1 Bioconversion Laboratory, Institute for Problems of Chemical and Energetic Technologies, Siberian Branch of the Russian Academy of Sciences (IPCET SB RAS), Biysk 659322, Russia; 32nadina@mail.ru (N.A.S.); evg-gladysheva@yandex.ru (E.K.G.); zenkova_nastasya080401@mail.ru (A.A.Z.); 2 Department of Biotechnology, Biysk Technological Institute, Polzunov Altai State Technical University, Biysk 659305, Russia

**Keywords:** *Miscanthus × giganteus*, sustainable development, enzymatic hydrolysis, bacterial nanocellulose, SCOBY, chemical pretreatment of biomass

## Abstract

The concept of bacterial nanocellulose (BNC) production from low-cost cellulosic raw materials is evolving across the world, as it reduces the production cost of this valuable polymer and expands its technical applications. *Miscanthus × giganteus* is a widely recognized energy crop with high cellulose content, but its potential as a feedstock for BNC production is underexplored. The cellulose content in the biomass of *Miscanthus × giganteus* from the Russian breeding stock was 54% in the present study. The *Miscanthus × giganteus* biomass was subjected to chemical pretreatment by four different techniques: classical alkaline delignification and three authors’ own methods using diluted nitric acid solutions at atmospheric pressure. The resultant substrates were then enzymatically hydrolyzed under identical conditions, yielding carbohydrate-based culture media on which bacterial nanocellulose biosynthesis was carried out using a SCOBY symbiotic culture. All the four chemical pretreatment methods were found to be extremely efficient because they provide a 28–31-fold increase in the biomass reactivity to enzymatic hydrolysis compared to untreated *Miscanthus × giganteus*. This study clearly demonstrates that it is most expedient to carry out the biomass pretreatment in a single stage using a dilute nitric acid solution in the BNC production technology from *Miscanthus × giganteus*. In this case, the substrate yield from the feedstock for subsequent hydrolysis was 50%, the recovery of reducing sugars from the *Miscanthus × giganteus* biomass reached its maximal value (65.2%), and the yield of BNC was 1.1–1.3 times higher compared to the other three methods of biomass pretreatment.

## 1. Introduction

Bacterial nanocellulose (BNC) is a unique microbial polymer which, thanks to its nanoscale size and chemical purity, possesses properties that differ from any plant-based cellulose, and has a wide range of applications. The bibliometric analysis of BNC has revealed a growing interest in this material among specialists in various fields. Biomedical research remains the primary focus of scientific research, while a new trend is the creation of new composites for technical applications [1,2,3].

The industrial sectors, in which cellulose has traditionally been used but which have undergone significant transformation with the advent of BNC, include the pulp and paper industry [4,5], consumer goods industry [6,7,8,9], and chemical industry. The incorporation of BNC into these traditional industries opens up new horizons, as the properties of the products obtained from BNC differ significantly from those of the conventional products. For example, BNC nitrates and their composite explosives were obtained [10,11].

The new application areas of cellulose, more specifically BNC, or more precisely BNC-based composites, are the biotechnology industry [5,12,13] (composite membranes for the separation of cells, viruses, macromolecules; or as a matrix for the immobilization of cells, tissues, molecules) and a related area like the environmental protection, where the composites are used as pollutant sensors or directly for the wastewater treatment and bioremediation [14,15].

BNC has revolutionized industrial sectors such as power engineering and electronics. In power engineering, BNC is used to make no-analog electrodes, supercapacitors, and fuel cells [15,16]. In electronics, the intensive development areas include the development of biosensors, carbon nanotubes, and flexible, wearable, flat-panel 3D electronics (solar cells, acoustic diaphragms, displays) [5,17,18].

The list of applications for BNC is expanding every year, and it is predicted that BNC-based composites will be expelling the conventional materials in mechanical engineering and related industrial sectors [15].

The demand for technical BNC exceeds supply manifold, but only a few groundbreaking scientific results have been implemented in industry. First of all, this is associated with the high production cost of BNC.

The metabolic features of the BNC microbial producers are such that their yield is not high and their cost is significant, so one of the ways to reduce the production cost is to use low-cost raw materials. Numerous studies have been focused on cheapening the nutrient media for BNC biosynthesis [7,19,20,21]. Therefore, the concept of transforming cheap cellulosic raw materials into expensive BNC is being actively developed around the world [7,19,20].

This area is of particular importance for the northern nations, where the growing season is very short and there is no possibility of harvesting sugar-rich biomass within that time; even harvesting starchy biomass is questionable. In such conditions, and with the global agenda in mind, the competition for food-grade raw materials between people and industrial enterprises is unacceptable; therefore, the concept of converting low-cost cellulosic raw materials into high-margin products matters not only within the context of the circular economy but also holds acute social significance [7,19,20,22,23,24]. We believe that it is unacceptable to produce technical bacterial nanocellulose (BNC) from food-grade feedstocks.

An important aspect is the adequate choice of cellulosic feedstock. *Miscanthus × giganteus*—an energy crop—occupies one of the leading positions in the world among cellulosic raw materials. The scale of the *Miscanthus × giganteus* plantings is increasing annually worldwide, thanks to its high growth rate, cellulose content higher than that of wood (50–55% versus 35–50%), and lower breeding costs. The use of *Miscanthus × giganteus* contributes to the formation of a new carbon-neutral bioeconomy across areas such as organic farming, construction, power engineering, pulp and paper industry, chemical industry, and industrial biotechnology [25,26,27,28].

Biotechnological transformation of plant raw materials is one of the most promising industrial areas, allowing high-value products to be obtained from the available, low-cost plant feedstocks [29,30,31]. At the same time, there are few examples of obtaining BNC from *Miscanthus* in the global practice [32,33], and there is no comprehensive assessment of the technological cycle from raw materials to the final product.

Just like any other type of cellulosic feedstock, *Miscanthus × giganteus* consists of cellulose, hemicellulose, lignin, fat–wax fraction, and mineral components, which are tightly bound together into a composite matrix. According to the unanimous opinion of the world experts, the pretreatment of cellulosic raw materials is the key stage that determines the success of the subsequent stages of enzymatic hydrolysis and microbiological biosynthesis of any products of biotechnological transformation [29,30,31].

This is the first study that uses the authors’ own methods by the chemical pretreatment of *Miscanthus × giganteus* with nitric-acid solutions [34]. The distinctive feature is that the pretreatment was carried out at atmospheric pressure. Classical delignification with sodium hydroxide was also carried out herein at atmospheric pressure as the control pretreatment.

This study has calculated the yields of intermediate products and the target product using the four methods for chemical pretreatment of *Miscanthus × giganteus*, and evaluated the efficiency of the complete cycle of BNC biosynthesis from *Miscanthus × giganteus*, starting from the feedstock to the final product.

## 2. Materials and Methods

### 2.1. Feedstock and Pretreatment

*Miscanthus × giganteus* var. KAMIS from the Russian breeding stock was grown in the village of Marushkino, Moscow Oblast, and was provided by OOO “Master Brand” (Moscow, Russia).

This paper examined four pretreatment methods of *Miscanthus × giganteus* using 4 wt.% dilute solutions, i.e., one-stage pretreatments with a nitric acid solution (Substrate 1) or a sodium hydroxide solution (Substrate 2) and two-stage pretreatments with a nitric acid solution first and then with a sodium hydroxide solution (Substrate 3) and vice versa―with a sodium hydroxide solution first and then with a nitric acid solution (Substrate 4). Nitric acid (CAS 7697-37-2, 99.0%) was procured from CHIMMEDSNAB (Korolev, Russia) and sodium hydroxide (CAS 1310-73-2, not less than 99.0%) from Scharlab (Sentmenat, Spain). All four pretreatment methods were carried out at atmospheric pressure under lab-scale conditions. The methods were previously described in detail [30,31]. These are authors’ own methods that have been repeatedly reproduced under laboratory and pilot conditions using feedstocks such as oat hulls and *Miscanthus sacchariflorus* var. Soranovskii. The successful pretreatment of *Miscanthus × giganteus* was achieved only after careful sample preparation that involved fivefold grinding on a KR-02 fodder chopper (Miass, Russia) to a size of no more than 12 mm, with 50% of the feedstock chopped to a size of no more than 4 mm. The chemical composition of the feedstock and pulps was determined by standard wet methods on a dry matter basis. The methods were previously described in [34].

### 2.2. Enzymatic Hydrolysis of Substrates from Miscanthus × giganteus

The obtained substrates were subjected to enzymatic hydrolysis with cellulolytic enzymes Cellolux-A (Sibbiofarm, Berdsk, Russia) and Ultraflo Core (Novozymes A/S, Bagsværd, Denmark), as reported in [30]. The multi-enzyme cocktail was added as follows: CelluLux-A 40 FPU/g solid and Ultraflo Core 15 FPU/g solid, and hydrolysis duration was 72 h. The peculiar feature of the present work is that it used a 0.05 M acetate buffer because we found that this concentration has a favorable effect on the progression of enzymatic hydrolysis and is not inhibitory to BNC biosynthesis.

### 2.3. Biotransformation of Hydrolytic Media into Bacterial Nanocellulose

The obtained enzymatic hydrolyzates were standardized against reducing sugars (RS, 20 g/L) and black tea extractives (1.6 g/L) and utilized as nutrient media for the biosynthesis of BNC using a symbiotic SCOBY (symbiotic consortium of bacteria and yeast, also known as Kombucha and *Medusomyces gisevii* Sa-12), purchased from the All-Russian Collection of Industrial Microorganisms (Pushchino, Moscow Region, Russia). We have previously demonstrated that SCOBY functions more effectively in hydrolytic media than individual strains of *Komagataeibacter xylinus* [35].

The seed material was pre-activated for 7 days under optimal conditions [36] and added to the hydrolytic medium at a concentration of 10 vol.%. The total cell count of yeast in the inoculum was 12.9–13.2 × 10^6^ cells per 1 cm^3^ and the total count of acetic bacteria was 1.6–2.2 × 10^6^ cells per 1 cm^3^. A synthetic glucose medium containing 20 g/L of glucose and 1.6 g/L of black tea extractives was used as the control [37]. BNC biosynthesis was carried out under optimal conditions identified previously [38]: static culture and a temperature of 27 °C. Culturing was carried out in a climate chamber (Binder, Neckarsulm, BW, Germany) for 11 days.

Upon completion of cultivation, a BNC gel-film was removed from the surface of the growth medium and washed from growth medium components and cells by stepwise treatment with 2 wt.% NaOH and 0.25 wt.% HCl (CAS 7647-01-0, 99.9%, OOO ALDOSA, Moscow, Russia), followed by washing with distilled water until neutral reaction. The resulting BC films were freeze-dried in an HR7000-M freeze-drier (Harvest Right LLC, Salt Lake City, UT, USA) to a constant weight, according to the reported procedure [39].

The yield of BNC (%) was calculated by the Formula (1):

(1) $$\eta =\;\frac{m_{BNC}}{C_{g}\cdot V\cdot 0.9}\cdot 100,$$

where *η* is the BNC yield, %; *m*_BNC_ is the BNC sample weight on an oven-dry basis, g; *C_g_* is the glucose concentration in the medium, g/L; *V* is the volume of the medium, L; 0.9 is the coefficient attributed to the water molecule detachment upon polymerization of glucose into cellulose.

### 2.4. Study of the BNC Structure

The degree of polymerization (DP) of BNC samples was measured by the viscosimetric method [40] using cadoxene as the solvent (ethylenediamine, CAS 107-15-3, not less than 99.0%, AO LenReaktiv, Moscow, Russia; cadmium oxide, CAS 1306-19-0, not less than 99.0%, OOO Khimreaktivsnab, Ufa, Russia).

For physicochemical studies, BNC samples were freeze-dried using an HR7000-M freeze-drier (Harvest Right, LLC., USA), according to the reported procedure [39].

The morphology of the BNC fiber surface was studied using scanning electron microscopy (SEM) on a GSM-840 scanning electron microscope (Jeol, Japan). For this, the freeze-dried samples were fixed on a conductive adhesive tape and sprayed with silver 10 nm thick for 2 min at 20–30 A before observation.

The thickness of the BNC fibers was measured using the Image J software (ver. 1.54p). 

Infrared spectroscopy was performed on an Infralum FT-801 ATR-FTIR spectrophotometer (OOO NPF Lumex-Sibir, Novosibirsk, Russia) at 4000–500 cm^−1^ using single and multiple attenuated total internal reflections.

Thermogravimetric analysis (TGA) was performed using a thermogravimetric analyzer (Shimadzu DTG-60 analyzer (Japan)). Experimental conditions: the sample was heated at a rate of 10 °C/min to a maximal temperature of 600 °C under nitrogen with a gas flow rate of 40 mL/min. The sample weight was P = 6.0–6.5 mg.

The experimental results were obtained in three repetitions and statistically processed by standard methods using Microsoft Office Excel 2019 software.

The work was carried out using equipment provided by the Biysk Regional Center for Shared Use of Scientific Equipment of the SB RAS (IPCET SB RAS, Biysk, Russia).

## 3. Results and Discussion

### 3.1. Analysis of the Compositional Profile of Miscanthus × giganteus and Its Pretreatment Products

**Figure 1:**
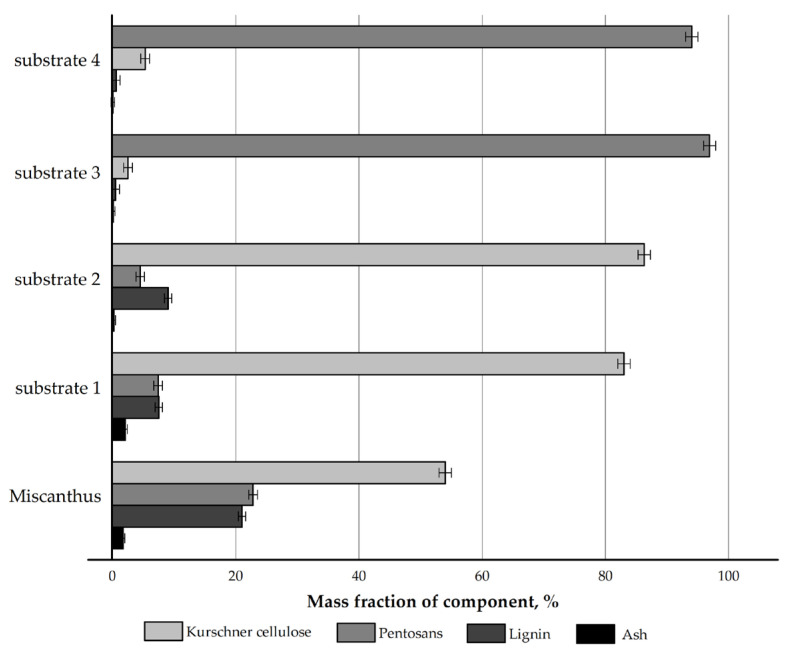
Compositional profile of *Miscanthus × giganteus* and its pretreatment products.

The chemical composition of the *Miscanthus × giganteus* biomass and its products obtained by four authors’ own pretreatment methods was initially examined. The results are depicted in Figure 1.

The pretreatment with nitric acid led to a 1.5-fold increase in the mass content of cellulose in the nitric-acid treatment product (substrate 1) (83.0% vs. 54.0%) compared to the feedstock and to a decrease in the content of non-cellulosics: pentosans by 3.1 times (7.4% vs. 22.8%) and lignin by 2.8 times (7.5% vs. 21.0%), while the proportion of mineral components increased from 1.7% to 2.1%, which is a feature of the nitric-acid treatment [34,36].

The pretreatment with sodium hydroxide was used as the reference method in this study, since it is regarded as a classical pretreatment method for non-woody cellulosic raw materials in the international community [30,31]. The cellulose content in the alkaline delignification product (substrate 2) increased 1.6-fold (86.3% vs. 54.0%). This method allows for the effective removal of pentosans, as their amount decreased 5.1-fold (4.5% vs. 22.8%). The residual lignin content was 9.0%, which is 2.3 times lower than that in the initial feedstock. The ash content during alkaline delignification decreased 8.9-fold (0.19% vs. 1.7%).

The pretreatment with nitric acid at the first stage and with sodium hydroxide at the second stage resulted in a chemically pure substrate—cellulose derived by the nitric acid method (substrate 3): the mass content of cellulose increased 1.8-fold (96.9% vs. 54.0%), the mass content of pentosans decreased 8.4-fold (2.5% vs. 21.0%), the mass content of lignin decreased 42-fold (0.5% vs. 21.0%), and the ash content decreased 17-fold (0.1% vs. 1.7%) as compared to the feedstock. This is a very effective method for cellulose isolation.

The pretreatment with sodium hydroxide at the first stage and with nitric acid at the second stage resulted in cellulose obtained by the modified alkaline method (substrate 4), i.e., a substrate with low ash content and low lignin content but with a residual pentosan content of 5.3%. This cannot be considered a disadvantage if the substrate is intended for hydrolysis to reducing sugars (RS).

The chemical compositions of the substrates obtained from *Miscanthus × giganteus* in this study are very close to those of the substrates derived from *Miscanthus sacchariflorus* in [34]. The reproducibility of the results indicates the stable performance of the authors’ own pretreatment methods for non-woody cellulosic raw materials and confirms their effectiveness.

### 3.2. Enzymatic Hydrolysis of Substrates from Miscanthus × giganteus

**Figure 2:**
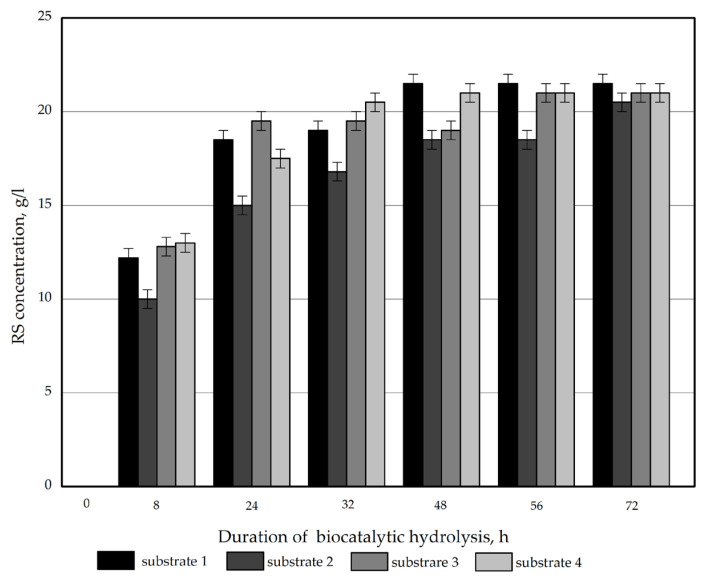
RS concentration plotted versus the time of enzymatic hydrolysis of the *Miscanthus × giganteus* substrates.

The concentration of reducing sugars (RS) plotted against on the hydrolysis time of the obtained samples is displayed in Figure 2.

All the samples were found to have a similar reactivity to enzymatic hydrolysis. The RS concentration after 72 h was 20.8–21.5 g/L. The RS yield on a substrate weight basis ranged from 63.0% (for substrate 2) to 65.2% (for substrate 1) for the obtained products. The obtained values are lower than those reported in relevant studies for *Miscanthus sacchariflorus* [30].

Table 1 summarizes data on RS yields resulting from the enzymatic hydrolysis of four pretreatment products from the *Miscanthus × giganteus* biomass, as well as the hydrolysis results for the untreated biomass.

All of the pretreatment types proved to be extremely effective for *Miscanthus × giganteus*, as they led to a 28–31-fold increase in the reactivity of hydrolyzables contained in the substrates (the sum of cellulose and hemicellulose (pentosans)) compared to the untreated feedstock: the RS yield ranged from 63.4% to 71.4% for the substrates vs. 2.3% for untreated *Miscanthus*. In the literature, pretreatment is considered successful if it leads to an increase in the substrate reactivity by a factor of 5 or more as compared to the feedstock [29]; this way, the results of the pretreatments used herein can be considered outstanding.

However, for scientific objectivity, it is necessary to note the extreme recalcitrance of untreated *Miscanthus × giganteus* to enzymatic hydrolysis: the PS yield was only 2.3% of the total hydrolyzables, which is 7.4 times lower than the hydrolyzability of untreated *Miscanthus sacchariflorus*, for which the RS yield is 17% [34]. This can be explained by the morphological features of *Miscanthus × giganteus*. *Miscanthus × giganteus* is a powerful, durable, strong plant and is twice as tall as *Miscanthus sacchariflorus*, with its stems being 2–3 times thicker than those of *Miscanthus sacchariflorus*. The morphological features also influenced the behavior of the substrates during enzymatic hydrolysis. Despite the significant increase in the reactivity to enzymatic hydrolysis following pretreatment, it is 13–25% lower for the *Miscanthus × giganteus* substrates than for the *Miscanthus sacchariflorus* substrates. This is a very important point. The obtained results are on a par with the literature sources [41,42], which note the exceptional resistance of *Miscanthus* to enzymatic hydrolysis, while the enzymatic hydrolysis behavior of natural substrates, rather than the model ones is primarily explained by the substrate morphology [42].

The substrates from *Miscanthus × giganteus* exhibit fairly high hemicellulose hydrolysis efficiency. The xylose yield on a pentosan content basis in the substrates ranges from 62.5% to 93.0% (Table 1). At the same time, the contribution of xylose to the total RS is only 2.4–9.3%; thus, the obtained hydrolyzates are mainly glucose ones.

### 3.3. Biotransformation of Hydrolytic Media into Bacterial Nanocellulose

At the third stage of the experiment, BNC biosynthesis was carried out on four hydrolyzates obtained. Table 2 shows the main biosynthesis process parameters.

The microbial producer used in this work is a consortium of various species and genera of yeast and acetobacteria. It is known from the literature that yeast synthesizes ethanol to stimulate the growth of acetobacteria, which in turn produce BNC to protect yeast from environmental exposure [43]. Therefore, the count of yeast exceeds that of acetobacteria. It should be noted that the contents of yeast and acetobacteria on different nutrient media differs slightly: upon completion of cultivation, the count of yeast cells ranged from 14 to 18 (20 in the control) × 10^6^ cells per 1 mL of the medium, while the acetobacteria count ranged from 8 to 13 (15 in the control) × 10^6^ cells per 1 mL of the medium. The decrease in pH during cultivation occurs due to the active vital activity of acetobacteria and due to the formation of metabolites such as acetic acid, succinic acid, gluconic acid, etc. [44]. This creates favorable conditions for the vital activity of the microbial producer and reduces the risk of contamination by foreign microflora. In this case, it should be noted that the initial acidity of all four substrates was already in the acidic range (4.3–4.5 pH), in contrast to the control medium, which initially had a neutral pH value (7.0). Upon completion of cultivation, the acidity of the nutrient media prepared from the experimental substrates declined to 3.2–3.8 pH (compared to 2.9 pH in the control). The residual RS concentration at 11 days of cultivation was minimal in the synthetic nutrient medium (control) and amounted to less than 1 g/L. The RS concentration in the nutrient media from substrate 1, substrate 3 and substrate 4 ranged from 3.2 to 5.2 g/L. The highest residual RS concentration of 8 g/L was observed in the medium from substrate 2. The same nutrient medium had the lowest contents of yeast and acetobacteria, which is explained by the adverse effect of sodium ions on the biosynthesis of BNC [45].

The most significant indicator of the process efficiency is the yield of the target product. When it comes to BNC, there are two illustrative ways of results representation: calculation of the BNC yield on a RS content basis and on an initial feedstock basis. The highest BNC yields were obtained on the nutrient media prepared from the enzymatic hydrolyzates—substrate 3 and substrate 4 (10.7% and 10.4%, respectively)—and were comparable to the control (11.8%). For hydrolyzate substrate 1, the yield was 8.7%, and the lowest BNC yield was obtained on the medium from substrate 2 and was 7.8%. In a similar study in which Kombucha Original Bio was used as the microbial producer, the BNC yield ranged from 1 to 4%, which is 3.0–7.8 times lower than those obtained in the present study [46].

**Figure 3:**
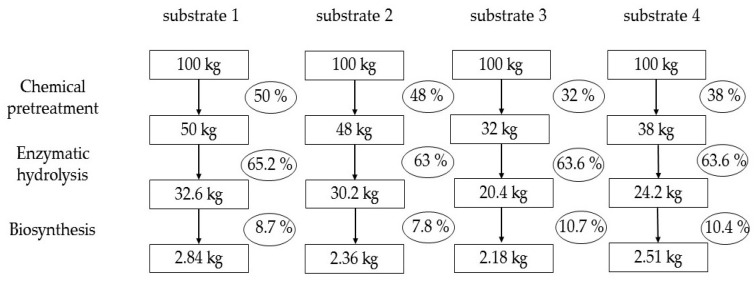
Calculation of the yields of intermediate products and of the target product (BNC) from *Miscanthus × giganteus* (calculated on an oven-dry basis).

Figure 3 summarizes the yield calculation data for intermediate products and BNC from *Miscanthus × giganteus*. This is a technological calculation; therefore, for the enzymatic hydrolysis stage, the RS yield is expressed on a substrate weigh basis rather than on a hydrolyzables content basis, in order to objectively assess what product yield will be in real production. The BNC yield is also expressed on a total RS basis in the substrates rather than on a utilized RS basis in order to avoid mathematical overestimation of the yield. The modern researchers who investigate BNC biosynthesis often mathematically overestimate the yield [47,48], unlike the classical works that adhere to real calculations [49,50].

Analysis of Figure 3 shows that the pretreatment makes the most significant contribution, as it determines the yield of substrates on a *Miscanthus* weight basis. This is a key stage of the technology, which has the greatest impact on the overall yield of BNC. At the enzymatic hydrolysis stage, approximately equal yields of RS were obtained on a substrate weight basis. At the BNC biosynthesis stage, the highest yields of 10.7 and 10.4% were obtained for substrates 3 and 4, respectively, the technological feature of which is the two-stage pretreatment of *Miscanthus × giganteus*. The classic pretreatment with sodium hydroxide (substrate 2) resulted in a biologically poor-quality nutrient medium and, consequently, in a low BNC yield of 7.8%. We believe that this is due to the presence of residual sodium ions, which are very difficult to wash out of the substrate [30].

Only a comprehensive analysis made it possible to determine the leader. Obviously, the pretreatment with nitric acid proved to be the most effective, providing a 2.84% yield of completely dry BNC on a *Miscanthus* weight basis, which is 1.1–1.3 times higher than that obtained using the other pretreatment methods.

In the present study, the degree of polymerization (DP) of the obtained BNC samples was also determined and was 2500 for the synthetic nutrient medium (control), 2830 for substrate 1, 2600 for substrate 2, 3050 for substrate 3, and 3000 for substrate 4. The obtained values are close to each other and quite high.

The study [51] investigated the synthesis of BNC using alternative carbon substrates, namely waste and byproducts from the biodiesel and confectionery industries (crude glycerin, sunflower seed meal hydrolyzates, pastries waste hydrolyzates). The BNC samples we obtained had a degree of polymerization ranging from 1889.1 to 2672.8, which is lower than in [51]. During the biosynthesis of BNC on media using various pure sugars as a carbon source [52], which can be considered ideal conditions, the highest degree of polymerization was observed on media with glucose and maltose (4350–4400), and the lowest one on media with mannose (2340). Thus, BNC samples obtained from *Miscanthus × giganteus* in hydrolytic media have degrees of polymerization that are quite comparable to those of BNC samples obtained from both pure sugar media and media with alternative carbon sources.

### 3.4. Comparison Between Conventional Production Method and Authors’ Production Method for BNC from Miscanthus × giganteus

According to [7], one of the world’s largest producers of BNC is Hainan Yeguo Foods Co., Ltd. (Hainan, China) where BNC is produced from low-cost food raw materials that do not require chemical pretreatment: coconut water, corn cobs, alcohol waste, pineapple peel, citrus juice, and apple juice. Static fermentation using mesophilic strains of *G. xylinus* is used. The produced BNC is intended for the food industry.

The main advantage of the developed method for obtaining BNC from *Miscanthus × giganteus* is the use of cellulosic raw materials. This is very important for countries in the northern hemisphere, where climatic conditions are such that it is impossible to have sugar-containing raw materials grown within the vegetational season. *Miscanthus × giganteus* is naturally resistant to microbial spoilage [53] and, consequently, is in well storage, which is very important for the stable operation of a biorefinery.

In terms of pricing, for example, on alibaba.com (a globally accessible marketplace), the crushed *Miscanthus* is being sold at US $5.76 per 25 kg or at US $0.23 per kg (La Vague Eco, Trébédan, France) [54], while glucose is being marketed at US $1.0 per kg (Guangzhou ZIO Chemical Co., Ltd., Guangzhou, China) [55].

It is clear that the major costs for the BNC production will be for the sugar solution from *Miscanthus × giganteus*. Here, we encounter limitations that are well described for any cellulose-containing raw materials. These limitations mean that the nutrient media from the hydrolyzates are more expensive than the food media, as is the case, for example, at Hainan Yeguo Foods Co., Ltd.

First, this is not the cellulosic feedstock recalcitrance that can be fixed by its pretreatment. In our case, the reactivity of *Miscanthus × giganteus* to enzymatic hydrolysis after the one-stage pretreatment with the dilute nitric-acid solution increases 31-fold. This is an outstanding outcome.

Second, this is the enzymatic hydrolysis efficiency. In our case, it was 65.2%, which is an acceptable level, but it can be significantly increased by using more effective enzymes [56,57].

Third, it is the comprehensive use of cellulose-containing raw materials. Cellulose, hemicellulose, and lignin must all be commercially marketed [29,56]. Our technology meets this criterion, as cellulose is converted into glucose and then into BNC, while hemicellulose and lignin pass to the spent nitric acid solution and constitute a combined lignin-humic fertilizer [58]. The sale of the fertilizer will compensate for the costs for pretreatment and enzymatic hydrolysis.

Thus, the main limitations associated with the use of the cellulosic feedstock in this work have been overcome, or there is a roadmap for overcoming them (in terms of the efficiency of enzymatic hydrolysis).

### 3.5. Study of the BNC Structure

**Figure 4:**
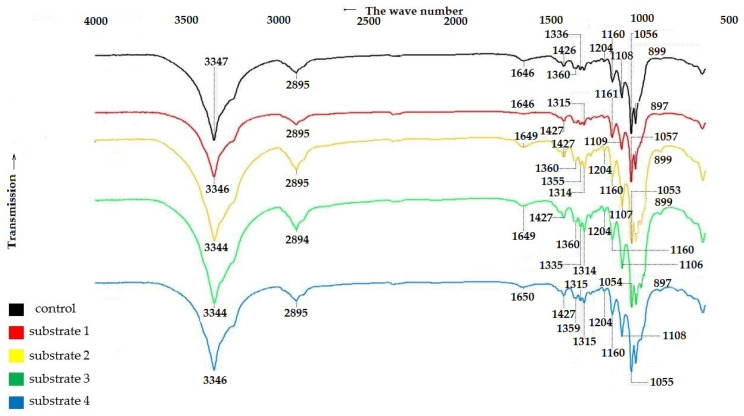
IR spectra of BNC samples obtained from *Miscanthus × giganteus*.

To confirm the structure of the BNC samples, IR spectra of the control and experimental BNC samples were recorded. The results are shown in Figure 4.

The IR spectroscopy data in Figure 4 show that all BNC samples, regardless of the pretreatment method of the feedstock, exhibit characteristic bands that are comparable to those reported the literature for BNC [59,60,61].

The broad band near 3344–3347 cm^−1^ corresponds to the stretching vibrations of OH groups, indicating the hydrophilic nature of the materials. The peak near 2894–2895 cm^−1^ corresponds to the stretching vibrations of C–H and CH_2_ groups, while the vibrations near 1646–1650 cm^−1^ are due to the bending vibrations of absorbed water molecules [59]. The IR spectra of BC samples are characterized by a well-defined structure of bands near 1426–1427 cm^−1^ and 1359–1360 cm^−1^, corresponding to the bending vibrations of the CH_2_ group and CH group [60,62]. The absorption bands at 1335 cm^−1^ and 1315 cm^−1^ are characteristics of the bending vibrations of OH groups in alcohols. The bending vibrations at 1279–1280 cm^−1^ correspond to the CH_2_ vibrations in alcohols, and those at 1204 cm^−1^ correspond to the bending vibrations of water. The bands located near 1160–1161 cm^−1^, 1106–1109 cm^−1^ and 1053–1057 cm^−1^ are due to the asymmetric stretching of the bridge of the C–O–C b-glycosidic bond, stretching of the C–O skeletal bonds of the pyranose ring, and stretching of the C–O bonds of the cellulose molecule [62].

The band near 897–899 cm^−1^ corresponds to the first carbon atom involved in the formation of the β-glycosidic bond [61].

A comparison of the characteristic frequencies of the IR spectra of the BNC samples shown in Figure 1 revealed that all the samples, regardless of the growth medium used, are characterized by close values of stretching vibrations corresponding to cellulose vibrations. This evidences that the samples have the same polymer structure [59,60,61].

**Figure 5:**
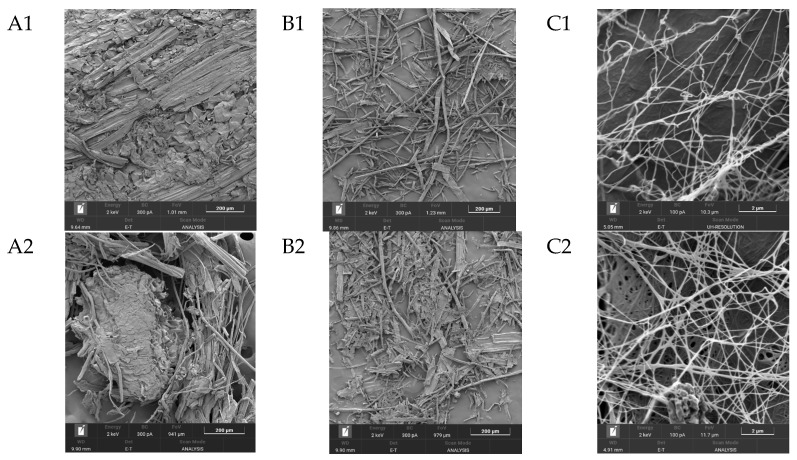

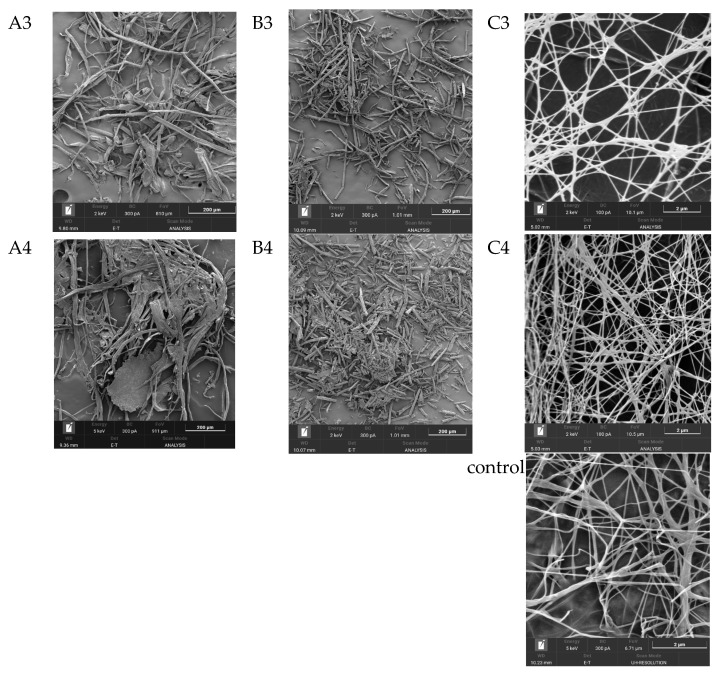
SEM results (zoom × 5000): (**A1**–**A4**), substrate samples (substrates 1–4) before enzymatic hydrolysis; (**B1**–**B4**), substrate samples (substrates 1–4) after enzymatic hydrolysis; (**C1**–**C4**), BNC samples synthesized on hydrolyzates obtained from these substrates; and the control, a BNC sample obtained on a synthetic nutrient medium.

One of the most important characteristics of BNC, which largely determines its properties, is its unique three-dimensional nanostructure. This structure represents intertwined, extremely thin cellulose fibers, which are nearly 100 times thinner than plant cellulose fibers and form a strong three-dimensional network through hydrogen bonds [63]. Figure 5 shows SEM images of the substrates from *Miscanthus × giganteus* (substrates 1–4) before and after enzymatic hydrolysis, as well as SEM images of BNC samples obtained from these substrates.

The minimal width of microfibrils for the BNC samples, regardless of the pretreatment method, ranged from 34.0 to 42.0 nm, and the maximal width ranged from 151 to 162 nm. Thus, the average width of microfibrils for the BNC samples varied from 56.0 to 88.0 nm. The width of microfibrils for the BNC sample obtained on a synthetic nutrient medium (control) was slightly smaller and ranged from 24.0 to 105.0 nm. The average width of microfibrils was 56.0 nm. It is known from the literature that the width of BNC microfibrils may depend either on the nature of the microbial producer or on the nutrient medium composition [64,65,66]. Based on the results obtained in this study, it can be concluded that the width of BNC microfibrils does not depend on the pretreatment method of the *Miscanthus × giganteus* biomass.

**Figure 6:**
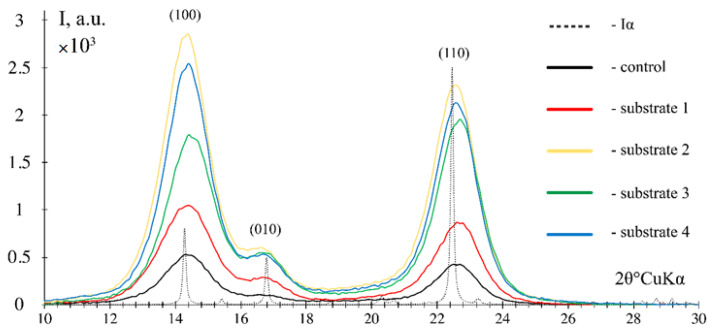
X-ray diffraction images of the BNC samples obtained from *Miscanthus × giganteus* in reflection geometry.

Figure 6 shows the X-ray diffraction data for the obtained BNC samples.

The X-ray diffraction images of the BNC samples obtained from enzymatic hydrolyzates of *Miscanthus × giganteus* substrates revealed three characteristic diffraction peaks at 14.5 ± 0.5, 16.5 ± 0.5 and 22.5 ± 0.5, corresponding to the cellulose structure and literature data [67,68]. The experimental X-ray diffraction patterns in reflection geometry are consistent with the theoretical curve constructed for the Iα cellulose model. The calculation using full-profile analysis showed that in all BNC samples obtained, the triclinic phase Iα accounts for 100%. The prevailing triclinic phase Iα in the BNC samples is recognized by the scientific community [69]. The BNC index of crystallinity was as follows: 78% on a synthetic nutrient medium (control), 85% on substrate 1, 83% on substrate 2, 82% on substrate 3, and 84% on substrate 4. The values obtained are close and high, and are confirmed by the literature data [68,70]. It is known that when the nutrient medium for BNC biosynthesis is replaced, the index of crystallinity may decline and the ratio between Iα and Iβ phases may change. For instance, as reported in [39], when the culture medium was replaced by culture media prepared from food industry byproducts, the index of crystallinity decreased from 53% to 26%, and the proportion of the monoclinic phase Iα decreased from 96% to 61%; similar data were obtained in a relevant study [71]. In the present study, the use of *Miscanthus × giganteus* biomass-derived hydrolyzates obtained after various chemical pretreatment methods did not lead to a significant change in structural characteristics, which is due to the nature of the SCOBY producer used. This microbial producer having high stress resistance [72] showed stable structural characteristics of BNC when different nutrient media were employed [36,73].

**Figure 7:**
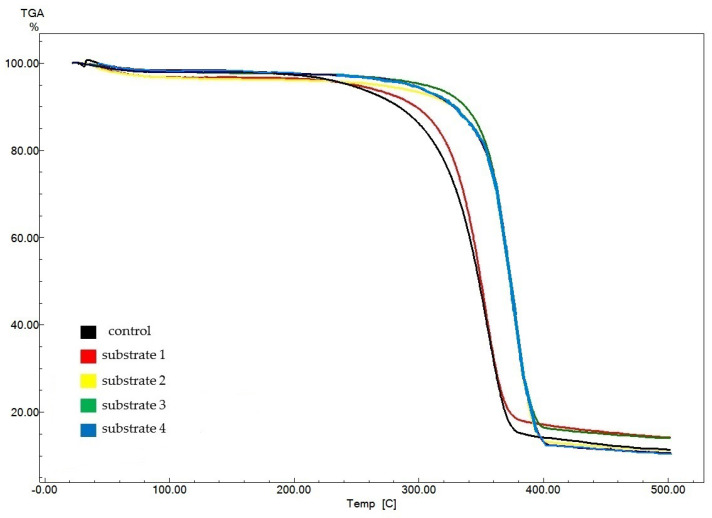
TGA results for BNC samples obtained from *Miscanthus × giganteus*.

Figure 7 displays thermogravimetric analysis (TGA) data for experimental BNC samples (obtained from substrates 1–4) as compared to data for the control sample obtained on a synthetic nutrient medium.

The control sample showed the lowest onset temperature of pyrolysis (309 °C), which characterizes the thermal stability of the samples. For the experimental samples, the pyrolysis onset temperature ranged from 318 °C (for the BNC sample obtained on substrate 1) to 355 °C (for the sample obtained on substrate 2). The amount of residues after decomposition of the BNC samples ranged from 10.4% (for the BNC sample obtained on substrate 4) to 14.2% (for the BNC sample obtained on substrate 1). The obtained data suggest an identical structure and high purity of the BNC samples. The obtained TGA data are in good agreement with those reported in the world scientific literature [74,75,76,77,78,79,80,81].

Summarizing the results presented in this paper and comparing them with published scientific data, it can be noted that the yield of BNC from *Miscanthus × giganteus* is 1.6–1.8 times higher than that of BNC from *Miscanthus sacchariflorus* [36]. Since the same methods and approaches were used in both cases, the differences in yields are due to the characteristics of the feedstocks. The chemical compositions of *Miscanthus × giganteus* and *Miscanthus sacchariflorus* are very similar, but it is likely that *Miscanthus sacchariflorus* contains minor components that are responsible for its winter hardiness and resistance to external factors, and it is these components that are also inhibitory to BNC biosynthesis. BNC microbial producers are known to be sensitive to inhibitors and to be demanding of the composition of nutrient media [19,20,82]. An important aspect is the reproducibility of results: both in [36] and in the present work, the maximal BNC yield is achieved by the single-stage pretreatment of the *Miscanthus × giganteus* biomass with nitric acid.

In [32], *Miscanthus* (the species was not specified) was subjected to hydrothermal treatment in the presence of sulfuric acid, the resulting pulp was then subjected to enzymatic hydrolysis using the Celic CTec2 enzyme (Novozymes), the hydrolyzate was standardized against RS (50 g/L), nutrients and vitamins were added, and BNC was then synthesized using *Gluconacetobacter xylinus* ATCC 53524, with a productivity of 16.7 g/L. Thus, the biosynthesis stage was carried out with a high yield (37% of the RS concentration), but, unfortunately, those authors did not provide a material balance for the BNC production from *Miscanthus*; therefore, it is not possible to compare the yields.

## 4. Conclusions

This study unveils the considerable potential of the low-cost energy crop *Miscanthus × giganteus* for producing high-value BNC biopolymer. The complete technological cycle of converting *Miscanthus × giganteus* into BNC includes (1) chemical pretreatment with dilute nitric acid solutions at atmospheric pressure in comparison with classical delignification; (2) enzymatic hydrolysis of the resulting substrates; (3) preparation of a nutrient medium based on enzymatic hydrolyzates; (4) biosynthesis of BNC using a SCOBY; and (5) purification of BNC.

The pretreatment makes the most significant contribution to the complete cycle, as it determines the yield of substrates on a *Miscanthus* weight basis and the BNC yield in the overall process. The most effective method for pretreatment of *Miscanthus × giganteus* is a one-stage pretreatment with nitric acid, in which case the substrate yield was 50% and the BNC yield was 2.84% on a *Miscanthus* weight basis.

Despite the recalcitrance of pristine *Miscanthus × giganteus* to enzymatic hydrolysis, all four pretreatment methods are extremely effective, as they provide a 28–31-fold increase in the reactivity to enzymatic hydrolysis compared to untreated *Miscanthus × giganteus*. The lower the content of non-cellulosic impurities in the substrate, the higher the biological quality of the nutrient medium and the higher the BNC yield at the biosynthesis stage.

In all four cases, the samples were pure cellulose (from the IR spectroscopic and thermogravimetric analysis data) and had a characteristic 3D nanostructure of fibers (SEM), and all the obtained BNC samples had 100% triclinic phase Iα (from the X-ray diffraction data).

## Figures and Tables

**Table 1:** Enzymatic hydrolysis of *Miscanthus × giganteus* substrates.

Pretreatment Method of *Miscanthus × giganteus*	RS Concentration in Hydrolyzate, g/L	RS Yield on a Basis of Total Cellulose and Total Pentosans in Substrate, %	Xylose Concentration in Hydrolyzate, g/L	Xylose Yield on a Pentosan Content Basis in Substrate, %	Contribution of Xylose to Total RS, %
substrate 1	21.5 ± 0.2	71.4 ± 0.3	2.0 ± 0.1	80.0 ± 0.2	9.3 ± 0.2
substrate 2	20.8 ± 0.2	68.6 ± 0.3	1.4 ± 0.1	93.0 ± 0.2	6.7 ± 0.2
substrate 3	21.0 ± 0.2	63.4 ± 0.3	0.5 ± 0.1	62.5 ± 0.2	2.4 ± 0.2
substrate 4	21.0 ± 0.2	67.1 ± 0.3	1.3 ± 0.1	72.2 ± 0.2	6.2 ± 0.2
untreated *Miscanthus × giganteus*	0.6 ± 0.1	2.3 ± 0.2	0.0	0.0	0.0

**Table 2:** Main parameters of the BNC biosynthesis process (before and after cultivation).

Nutrient Medium	Yeast Count (10^6^ Cells/mL)		Acetobacteria Count (10^6^ Cells/mL)		Acidity of the Medium, pH		RS Concentration, g/L		BNC Yield, %
	Before	After	Before	After	Before	After	Before	After	
Synthetic medium based on black tea (control)	15.0 ± 0.3	20.0 ± 0.3	9.0 ± 0.2	15.0 ± 0.3	7.0 ± 0.2	2.9 ± 0.2	20.0 ± 1.0	0.8 ± 0.7	11.8 ± 0.3
substrate 1		15.0 ± 04		9.0 ± 0.2	4.4 ± 0.1	3.2 ± 0.2	21.5 ± 0.7	3.2 ± 0.3	8.7 ± 0.3
substrate 2		14.0 ± 0.3		8.0 ± 0.3	4.5 ± 0.2	3.5 ± 0.1	20.5 ± 0.8	8.0 ± 0.3	7.8 ± 0.3
substrate 3		18.0 ± 0.4		13.0 ± 0.3	4.3 ± 0.2	3.5 ± 0.2	21.0 ± 0.8	4.0 ± 0.2	10.7 ± 0.3
substrate 4		17.0 ± 0.2		12.0 ± 0.2	4.4 ± 0.1	3.8 ± 0.2	21.0 ± 0.6	5.2 ± 0.5	10.4 ± 0.4

## Data Availability

The original contributions presented in this study are included in the article. Further inquiries can be directed to the corresponding author.
